# Post-operative sepsis-induced acute respiratory distress syndrome: risk factors for a life-threatening complication

**DOI:** 10.3389/fmed.2024.1338542

**Published:** 2024-03-05

**Authors:** Miguel Bardají-Carrillo, Marta Martín-Fernández, Rocío López-Herrero, Juan Manuel Priede-Vimbela, María Heredia-Rodríguez, Esther Gómez-Sánchez, Estefanía Gómez-Pesquera, Mario Lorenzo-López, Pablo Jorge-Monjas, Rodrigo Poves-Álvarez, Jesús Villar, Eduardo Tamayo

**Affiliations:** ^1^BioCritic, Group for Biomedical Research in Critical Care Medicine, Valladolid, Spain; ^2^Anesthesiology and Critical Care, Clinical University Hospital of Valladolid, Valladolid, Spain; ^3^Centro de Investigación Biomédica en Red de Enfermedades Infecciosas (CIBERINFEC), Instituto de Salud Carlos III, Madrid, Spain; ^4^Department of Medicine, Toxicology and Dermatology, University of Valladolid, Valladolid, Spain; ^5^Department of Surgery, University of Valladolid, Valladolid, Spain; ^6^Department of Anaesthesiology and Critical Care, Hospital Clínico Universitario de Salamanca, Salamanca, Spain; ^7^CIBER de Enfermedades Respiratorias, Instituto de Salud Carlos III, Madrid, Spain; ^8^Research Unit, Hospital Universitario Dr. Negrín, Las Palmas de Gran Canaria, Spain; ^9^Li Ka Shing Knowledge Institute at St. Michael’s Hospital, Toronto, ON, Canada

**Keywords:** ARDS, sepsis, septic shock, surgical patients, post-operative sepsis-induced ARDS

## Abstract

**Introduction:**

Prevalence and mortality of the acute respiratory distress syndrome (ARDS) in intensive care units (ICU) are unacceptably high. There is scarce literature on post-operative sepsis-induced ARDS despite that sepsis and major surgery are conditions associated with ARDS. We aimed to examine the impact of post-operative sepsis-induced ARDS on 60-day mortality.

**Methods:**

We performed a secondary analysis of a prospective observational study in 454 patients who underwent major surgery admitted into a single ICU. Patients were stratified in two groups depending on whether they met criteria for ARDS. Primary outcome was 60-day mortality of post-operative sepsis-induced ARDS. Secondary outcome measures were potential risk factors for post-operative sepsis-induced ARDS, and for 60-day mortality.

**Results:**

Higher SOFA score (OR 1.1, 95% CI 1.0–1.3, *p* = 0.020) and higher lactate (OR 1.9, 95% CI 1.2–2.7, *p* = 0.004) at study inclusion were independently associated with ARDS. ARDS patients (*n* = 45) had higher ICU stay [14 (18) vs. 5 (11) days, *p* < 0.001] and longer need for mechanical ventilation [6 (14) vs. 1 (5) days, *p* < 0.001] than non-ARDS patients (*n* = 409). Sixty-day mortality was higher in ARDS patients (OR 2.7, 95% CI 1.1–6.3, *p* = 0.024). Chronic renal failure (OR 4.0, 95% CI 1.2–13.7, *p* = 0.026), elevated lactate dehydrogenase (OR 1.7, 95% CI 1.1–2.7, *p* = 0.015) and higher APACHE II score (OR 2.7, 95% CI 1.3–5.4, *p* = 0.006) were independently associated with 60-day mortality.

**Conclusion:**

Post-operative sepsis-induced ARDS is associated with higher 60-day mortality compared to non-ARDS post-operative septic patients. Post-operative septic patients with higher severity of illness have a greater risk of ARDS and worse outcomes. Further investigation is needed in post-operative sepsis-induced ARDS to prevent ARDS.

## 1 Introduction

The acute respiratory distress syndrome (ARDS) is a life-threatening medical complication with a prevalence of about 10% in patients admitted to intensive care units (ICU), representing a 23% of patients requiring mechanical ventilation (MV) according to a recent international study (1), and with an associated hospital mortality of 40% (2). Regardless of its cause, ARDS may become a major ICU burden, as hospital stay and duration of MV are longer compared to other ICU patients (3), accounting for healthcare costs ranging from $54,490 to $450,888 in the US (4).

Sepsis is one of the most common causes of ARDS (5). Mortality among patients with severe sepsis and ARDS is up to fourfold higher than patients without ARDS (4). Surgery can also contribute to ARDS development, as post-operative ARDS represents a deleterious complication following major surgical procedures (6), being its incidence in the general surgical population approximately 0.2% (7). Post-operative sepsis is also a prevalent post-surgical complication, representing up to one-third of all septic patients (8) and being one of the major causes of death among surgical patients (9), being pneumonia after surgery the most frequent source of infection in post-operative patients (10). Therefore, post-operative sepsis-induced ARDS could be an important complication in ICU patients, as they are exposed to circumstances that favor ARDS development.

It has been reported that high APACHE II score, SOFA score, advanced age, shock, or urgent abdominal surgery are frequent conditions associated with ARDS development (4, 11), along with respiratory or abdominal infection as the most common sources of infection in ARDS patients (12). Furthermore, serum lactate and the presence of a microbiologically proven infection are independently associated with ARDS (4). However, no reliable system to identify patients at risk for developing ARDS has currently been developed (13). Once ARDS is in progress, only lung protective ventilation might prevent a fatal outcome (4).

Considering the 40% hospital mortality associated to ARDS (2), the higher mortality in septic patients that develop ARDS (4), and the scarce literature on post-operative sepsis-induced ARDS and its impact on mortality, we aimed to examine post-operative septic patients who met the Berlin criteria (14) for ARDS to evaluate its impact on 60-day mortality and to determine potential risk factors that could contribute to post-operative sepsis-induced ARDS prevention.

## 2 Materials and methods

### 2.1 Patient selection

This study is a secondary analysis of a prospective cohort of 454 adult (≥18 years old) patients undergoing major surgery, admitted to the surgical ICU of the 700-bed Hospital Clínico Universitario de Valladolid (Spain), between December 2006 and February 2017, who developed post-operative sepsis (15). All patients met the SEPSIS-3 criteria for sepsis or septic shock (16) and required invasive MV. The study protocol was approved by the Ethics Committee for Clinical Research, Hospital Clínico Universitario de Valladolid, Valladolid, Spain (approval No. PI 20-2070). This study complied with the current Spanish legislation for biomedical research and fulfilled the Declaration of Helsinki. Written informed consent was obtained from patients, patients’ relatives, or their legal representatives before enrolment.

We excluded patients who met clinical criteria for sepsis or septic shock, but had a negative microbiological culture. We also excluded patients on MV for <24 h, and patients with ARDS diagnosis prior to surgery. Included septic patients were stratified into two groups: (i) ARDS and (ii) non-ARDS (14). During surgery, patients’ ventilation was performed according to the attending clinicians, following a lung-protective ventilation protocol with a tidal volume of 6 to 8 ml per kilogram of predicted body weight, a PEEP of 6 to 8 cm of water, and recruitment maneuvers after tracheal intubation and repeated when necessary. Patients were managed according to current guidelines for general critical care management (16), which include: (i) early identification of causative microorganism, optimization of intravenous antibiotic selection and timely administration based on the antibiogram; (ii) fluid resuscitation and vasopressor use were individualized for maintaining a systolic blood pressure ≥90 mmHg or a mean arterial pressure ≥65 mmHg; and (iii) maintenance of hemoglobin between 7 and 10 g/dL according to the general clinical condition of patients (17). The choice of drugs for sedation and analgesia, hemodynamic treatment, and the decision to perform tracheostomy were left to the discretion of attending clinicians. Weaning from the ventilator was initiated when considered clinically appropriate by attending physicians. Gastric protection was routinely performed with omeprazole (20 mg/iv) during the first 24 h of ICU stay.

### 2.2 Data collection and follow-up

Patients admitted to the ICU were screened daily during the study period to assess the onset of sepsis/septic shock. We used a specific standardized form to collect demographic and clinical data, including hematological, biochemical, radiological, microbiological, and biomarker levels within the first 24 h after diagnosis of sepsis/septic shock. Disease severity was assessed using the Sequential Organ Failure Assessment (SOFA) scale (18) and the Acute Physiology and Chronic Health Evaluation II (APACHE II) (19) score. Sepsis was defined as a life-threatening organ dysfunction (indicated by an increase in SOFA score ≥2 points) resulting from an abnormal host response to infection (13). Septic shock was recognized by the need for a vasopressor to preserve a mean arterial pressure ≥65 mmHg and serum lactate >2 mmol/L (>18 mg/dL) in the absence of hypovolemia. After verifying that no patient was infected prior to the surgical procedure, we followed the criteria of the Centers for Disease Control and Prevention (CDC) (20) for diagnosis of nosocomial infections during ICU stay. ARDS was diagnosed according to Berlin definition (14) as: (i) hypoxemia occurring within 1 week of a well-known clinical insult or a further exacerbation of respiratory symptoms, (ii) bilateral opacities on chest radiographs that are not attributable to pleural effusions, lobar or pulmonary collapse, and (iii) acute respiratory failure not fully accounted either by cardiac insufficiency or fluid overload. A total of 60-day in-hospital mortality was assessed.

All clinical records were reviewed to identify patients meeting criteria for ARDS (14). Chest radiographs were assessed over the course of each patient’s ICU stay, while on MV for ≥24 h. For excluding patients with heart failure as a cause of pulmonary edema, echocardiographic images (when available), clinical history, or pulmonary arterial monitoring data were checked. Patients with dobutamine >5 mcg/kg/min or levosimendan infusion were excluded. PaO_2_/FiO_2_ ratios were collected at the time of ARDS diagnosis. Use of prone position was also checked in each patient.

### 2.3 Clinical endpoints and statistical analysis

The primary endpoint was 60-day mortality of ARDS. Secondary endpoints were potential risk factors for post-operative sepsis-induced ARDS development and for 60-day mortality.

Differences between groups were assessed using Chi-square test for categorical variables and the Mann Whitney *U*-test for continuous variables. Potential association between clinical variables and ARDS were evaluated using a Wald backward stepwise multivariate logistic regression analysis. Potential confounding factors for logistic regression were identified from variables described in Tables 1, 2, 4, 5. Variables yielding a *p*-value < 0.1 in the univariate regression analysis were included in the multivariate analysis as adjusting variables [cancer, napierian logarithm of PCT, napeirian logarithm of lactate and lactate dehydrogenase (LDH), gram-positive microorganism, abdominal infection, pneumonia, and septic shock]. We analyzed the probability of death to day 60 after sepsis diagnosis using Kaplan–Meier curves and tested with the log-rank test (Mantel–Haenszel).

**TABLE 1:** Preoperative and post-operative features based on ARDS.

	Non-ARDS (*n* = 409)	ARDS (*n* = 45)	*p*-value
**Characteristics**			
Age [years, median (IQR)]	72 (16)	76 (17)	0.096
Male [%, (*n*)]	61.1% (248)	57.8% (26)	0.667
**Comorbidities, [% (*n*)]**			
Chronic cardiovascular disease	33.6% (133)	22.2% (10)	0.123
Chronic respiratory disease	18.2% (72)	17.8% (8)	0.947
Arterial hypertension	59.1% (234)	57.8% (26)	0.865
Chronic renal failure	9.7% (38)	6.7% (3)	0.512
Diabetes mellitus	23% (91)	24.4% (11)	0.825
Cancer	32.6% (129)	46.7% (21)	0.059
Obesity	15.9% (63)	11.1% (5)	0.398
Smoker	16.9% (67)	20% (9)	0.604
**Surgery type, [% (*n*)]**			
Abdominal	83.1% (271)	92.8% (42)	0.103
Cardio-thoracic	4.3% (14)	0% (0)	0.171
Vascular	6.1% (20)	4.8% (2)	0.724
Urological/renal	3.1% (10)	0% (0)	0.250
Others	4.6% (15)	2.4% (1)	0.507
Urgent surgery	76.7% (297)	77.3% (34)	0.937
**Source of infection, [% (*n*)]**			
Respiratory tract	19.3% (79)	43.2% (19)	**0.007**
Abdomen	53.2% (202)	77.3% (34)	**0.002**
Urinary tract	4.5% (17)	2.3% (1)	0.491
Surgical site	1.3% (5)	0% (0)	0.443
Bacteremia	4% (15)	2.3% (1)	0.579
Others	12.9% (49)	6.8% (3)	0.243
**Microbiology [% (*n*)]**			
Gram +	31.3% (128)	11.1% (5)	**0.005**
Gram -	34.2% (140)	40% (18)	0.441
Fungi	13.4% (55)	15.6% (7)	0.696
**Severity scores and corticosteroids**			
SOFA score [median, (IQR)]	8 (5)	9 (2)	**0.005**
APACHE II score [median, (IQR)]	15 (7)	17 (5)	**<0.001**
Corticosteroids [%, (*n*)]	27.4% (112)	48.9% (22)	**0.003**
**Time course and outcomes**			
Length of MV (days) [median, (IQR)]	1 (5)	6 (14)	**<0.001**
Length of hospital stay (days) [median, (IQR)]	24 (24)	30 (27)	0.109
Length of ICU stay (days) [median, (IQR)]	5 (11)	14 (18)	**<0.001**
Septic shock [%, (*n*)]	74.1% (303)	93.3% (42)	**0.004**
Mortality at 60 days [%, (n)]	27.4% (112)	55.6% (25)	**<0.001**

Continuous variables are represented as median and interquartile range (IQR); categorical variables are represented as percentages (%) and number (*n*). SOFA, sequential organ failure assessment; APACHE, acute physiology and chronic health evaluation; MV, mechanical ventilation; ICU, intensive care unit. Bold values are statistically significant *p*-values.

**TABLE 2:** Measurements at diagnosis based on ARDS.

	Non-ARDS (1) (*n* = 409)	ARDS (2) (*n* = 45)	*p*-value
**Measurements at diagnosis, [median (IQR)]**			
Mean arterial pressure (mmHg)	68 (13)	63 (11)	0.259
Heart rate (bpm)	105 (30)	120 (38)	**<0.001**
PaCO2 (mmHg)	38.6 (11.1)	40 (13.8)	**0.030**
SaO_2_ (%)	97.2 (4.8)	94.4 (6.8)	**0.004**
PaO_2_ (mmHg)	100 (59.85)	82.9 (42.7)	**0.001**
pH	7.35 (0.13)	7.31 (0.15)	**0.015**
HCO_3_ (mmol/L)	21.5 (5.1)	21.5 (8.8)	0.749
FiO_2_ (%)	0.5 (0)	0.5 (0.2)	**0.002**
PaO_2_/FiO_2_ at study inclusion (mmHg)	244 (137)	133.7 (81.6)	**<0.001**
PaO_2_/FiO_2_ at 48 h (mmHg)	252 (133)	152 (99.5)	**<0.001**
Creatinine (mg/dl)	1.7 (2.3)	1.9 (1.9)	0.173
Lactate (mmol/L)	2.3 (2)	4.6 (3.3)	**<0.001**
Procalcitonin (ng/ml)	4.83 (18.1)	21.5 (58.4)	**0.011**
C-Reactive Protein (mg/L)	245 (150)	247.3 (241.9)	0.157
LDH (UI)	287 (250)	214 (177)	0.075

Continuous variables are represented as median and interquartile range (IQR). LDH, lactate dehydrogenase. Bold values are statistically significant *p*-values.

**TABLE 3:** Multivariate analysis for evaluating the risk of ARDS development.

	OR	[CI 95%]	*p*-value
Lactato Ln	1.905	1.205–2.703	**0.004**
SOFA	1.128	1.019–1.249	**0.020**

Bold values are statistically significant *p*-values.

**TABLE 4:** Preoperative and post-operative features based on 60-day mortality.

	Survivors 60 days (*n* = 317)	Non-survivors 60 days (*n* = 137)	*p*-value
**Characteristics**			
Age [years, median (IQR)]	71 (17)	76 (13)	**<0.001**
Male [%, (*n*)]	60.8% (192)	60.7% (82)	0.997
**Comorbidities, [% (*n*)]**			
Chronic cardiovascular disease	30.3% (93)	37.3 % (50)	0.147
Chronic respiratory disease	17.9% (55)	18.7% (25)	0.853
Arterial hypertension	56.7% (174)	64.2% (86)	0.141
Chronic renal failure	6.3% (20)	14.6% (20)	**0.005**
Diabetes mellitus	23.5% (72)	22.4% (30)	0.807
Cancer	34.2% (105)	33.6% (45)	0.899
Obesity	13.7% (42)	19.4% (26)	0.126
Smoker	17.6% (54)	16.4% (22)	0.764
**Surgery type, [% (*n*)]**			
Abdominal	86.7% (222)	78.6% (88)	**0.048**
Cardio-thoracic	3.9% (10)	3.6% (4)	0.877
Vascular	4.3% (11)	9.8% (11)	**0.040**
Urological/Renal	3.1% (8)	1.8% (2)	0.467
Other	3.1% (8)	7.1% (8)	0.082
Urgent surgery	78.7% (236)	72.5% (95)	0.164
**Source of infection, [% (*n*)]**			
Respiratory tract	19.3% (79)	43.2% (19)	**0.007**
Abdomen	56.8% (168)	53.1% (68)	0.490
Urinary tract	4.4% (13)	3.9% (5)	0.815
Surgical site	1.4% (4)	0.8% (1)	0.615
Bacteremia	3.7% (11)	3.9% (5)	0.930
Other	11.9% (35)	13.3% (17)	0.684
**Microbiology [% (*n*)]**			
Gram +	29.7% (94)	28.5% (39)	0.799
Gram -	35% (111)	34.3% (47)	0.884
Fungi	12.9% (41)	15.3% (21)	0.495
Gram – lung	5.7% (18)	11.7% (16)	**0.026**
Gram – routine surveillance	5.7% (18)	12.4% (17)	**0.014**
Fungi abdomen	2.5% (8)	6.6% (9)	**0.037**
**Severity scores and corticosteroids**			
SOFA score [median, (IQR)]	8 (6)	10 (4)	**<0.001**
APACHE II score [median, (IQR)]	14 (7)	17 (7)	**<0.001**
Corticosteroids	24% (76)	42.3% (58)	**<0.001**
**Time course and outcomes**			
Length of MV (days) [median, (IQR)]	1 (3)	4 (11)	**<0.001**
Length of hospital stay (days) [median, (IQR)]	26 (25)	21 (26)	**0.027**
Length of ICU stay (days) [median, (IQR)]	5 (9)	9 (14)	**<0.001**
Septic shock [%, (*n*)]	68.5% (217)	93.4% (128)	**<0.001**
ARDS [%, (*n*)]	6.3% (20)	18.2% (25)	**<0.001**

Continuous variables are represented as median and interquartile range (IQR); categorical variables are represented as percentages (%) and number (*n*). SOFA, sequential organ failure assessment; APACHE, acute physiology and chronic health evaluation; MV, mechanical ventilation; ICU, intensive care unit. Bold values are statistically significant *p*-values.

**TABLE 5:** Measurements at diagnosis based on 60-day mortality.

	Survivors 60 days (*n* = 317)	Non-survivors 60 days (*n* = 137)	*p*-value
**Measurements at diagnosis, median, (IQR)**			
Mean Arterial Pressure (mmHg)	70 (15)	65 (11)	**0.002**
Heart rate (bpm)	101 (30)	115 (35)	**<0.001**
Arterial SO_2_ (%)	97.2 (5.4)	96.4 (4.8)	0.464
Arterial PO_2_ (mmHg)	102 (59.4)	93.6 (56.0)	0.265
Arterial CO_2_ (mmHg)	37.9 (9.6)	40.75 (14.7)	0.061
Arterial pH	7.38 (0.12)	7.325 (0.15)	**<0.001**
HCO_3_ (mmol/L)	21.7 (4.5)	21.2 (6.4)	**0.011**
Venous pH	7.36 (0.10)	7.33 (0.14)	**0.004**
FiO_2_ (%)	0.5 (0.03)	0.5 (0)	**0.003**
PaO_2_/ FiO_2_ ratio (mmHg)	236 (139)	208 (140)	0.060
PaO_2_/FiO_2_ at 48 h (mmHg)	251 (147.8)	213.5 (119.5)	**<0.001**
Creatinine (mg/dl)	1.6 (1.8)	2.5 (2.8)	**<0.001**
Lactate (mmol/L)	2.2 (2.1)	3.7 (2.6)	**<0.001**
Procalcitonin (ng/ml)	4.5 (16.5)	7.8 (41.2)	**0.001**
C-reactive protein (mg/L)	251 (143)	231 (175.35)	0.872
LDH (UI)	242.5 (206)	313.5 (305)	**0.043**

Continuous variables are represented as median and interquartile range (IQR). LDH, lactate dehydrogenase. Bold values are statistically significant *p*-values.

We considered 2-sided *p*-values < 0.05 to indicate statistical significance. Statistical power was 99.9% with 95% confidence. All data were analyzed using the IBM SPSS 22.0 software (SPSS, Chicago, IL, USA).

## 3 Results

A total of 45 patients (9.9%) were diagnosed as having ARDS at study inclusion, whereas 409 (90.1%) patients did not develop ARDS. Baseline characteristics, at sepsis diagnosis, of patients are reported in Tables 1, 2, 4, 5. Patients developing ARDS were more likely to have septic shock [93.3%, (*n* = 42) vs. 74.1% (*n* = 303), *p* = 0.004] (Table 1) and were associated with higher SOFA [9 (2) vs. 8 (5), *p* = 0.005] and APACHE scores [17 (5) vs. 15 (7), *p* < 0.001] (Table 1), higher lactate [4.6 (3.3) vs. 2.3 (2.0), *p* < 0.001] and procalcitonin [21.5 (58.4) vs. 4.8 (18.1), *p* = 0.011] (Table 2). Prone position was not used in any patient. ARDS patients had lower PaO_2_ [82.9 (42.7) vs. 100 (59.9), *p* = 0.001] and arterial pH [7.31 (0.15) vs. 7.35 (0.13), *p* = 0.015], but also higher PaCO_2_ [40 (13.8) vs. 38.6 (11.1), *p* = 0.030], lower baseline PaO_2_/FiO_2_ [133.7 (81.4) vs. 244 (137.0), *p* < 0.001] and 48 h-PaO_2_/FiO_2_ [152 (99.5) vs. 252 (133.0), *p* < 0.001] (Table 2). ARDS patients had higher prevalence of pneumonia [43.2% (*n* = 19) vs. 19.3% (*n* = 79), *p* = 0.007] and abdominal infection [77.3% (*n* = 34) vs. 53.2% (*n* = 202), *p* = 0.002] (Table 1). Patients with ARDS had longer ICU stay [14 (18) vs. 5 (11) days, *p* < 0.001] and duration of MV [6 (14) vs. 1 (5), *p* < 0.001]. Mortality was higher in the ARDS group at day-60 (55.6% vs. 27.4%, *p* < 0.001) (Table 1). No patient required extracorporeal membrane oxygenation (ECMO).

Higher SOFA score (OR 1.1, 95% CI 1.0–1.2, *p* = 0.020) and higher lactate (OR 1.9, 95% CI 1.2–2.7, *p* = 0.004) were independently associated to ARDS development (Table 3).

Age was associated with mortality [76 (13) vs. 71 (17), *p* < 0.001], as previous chronic renal failure [14.6% (*n* = 20) vs. 6.3% (*n* = 20), *p* = 0.005] (Table 4). Respiratory tract infections [43.2% (*n* = 19) vs. 19.3% (*n* = 79), *p* = 0.007] and pulmonary gram-negative bacteria [11.6% (*n* = 16) vs. 5.7% (*n* = 18), *p* = 0.026] were more frequent in non-survivors. Abdominal fungi [6.6% (*n* = 9) vs. 2.5% (*n* = 8), *p* = 0.037] and gram-negative bacteria in routine cultures [12.4% (*n* = 17) vs. 5.7% (*n* = 18), *p* = 0.014] were also higher in non-survivors (Table 4).

Gas-exchange revealed that arterial pH was lower in non-survivors [7.33 (0.15) vs. 7.38 (0.12), *p* < 0.001]. PaO_2_/FiO_2_ at 48 h, but not at study inclusion, was lower in non-survivors [213.5 (119.5) vs. 251 (147.8), *p* < 0.001] (Table 5). In the non-survivors group, we found higher levels of lactate [3.7 (2.6) vs. 2.2 (2.1), *p* < 0.001], procalcitonin [7.8 (41.2) vs. 4.5 (16.5), *p* = 0.001] and LDH [313.5 (305) vs. 242.5 (206), *p* = 0.043] and creatinine [2.5 (2.8) vs. 1.6 (1.8), *p* < 0.001] (Table 5). Both SOFA [10 (4) vs. 8 (6), *p* < 0.001], and APACHE II [17 (7) vs. 14 (7), *p* < 0.001] scores, and use of corticosteroids during ICU stay [42.3% (*n* = 58) vs. 24% (*n* = 76), *p* < 0.001], as well as septic shock [93.4% (*n* = 128) vs. 68.5% (*n* = 217), *p* < 0.001] (Table 4) were higher in non-survivors.

**FIGURE 1:**
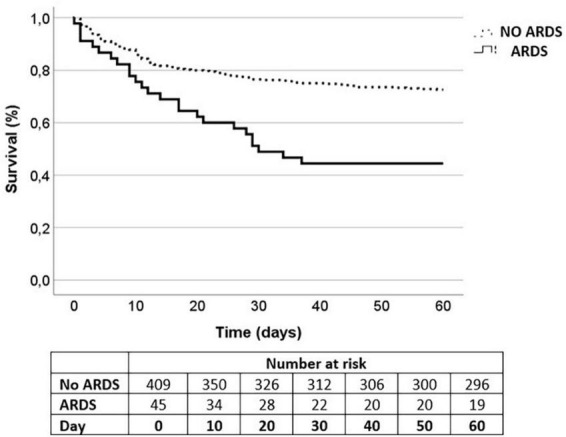
Kaplan-Meier survival curves for 60-day mortality.

Length of ICU stay [9 (14) vs. 5 (9) days, *p* < 0.001] and duration of MV [4 (11) vs. 1 (3) days, *p* < 0.001] was longer in non-survivors, and also the development of ARDS [18.2% (*n* = 25) vs. 6.3% (*n* = 20), *p* < 0.001] (Table 4). On average, patients who developed ARDS died 12 days earlier when assessing 60-day mortality (log-rank *p* < 0.001) (Figure 1). In the multivariate analysis (Table 6), ARDS was independently associated with 60-day mortality (OR = 2.7, 95% CI 1.1–6.3, *p* = 0.024), as well as APACHE II score >16 (OR = 2.7, 95% CI 1.3–5.4, *p* = 0.006), chronic renal failure (OR = 4.0, 95% CI 1.2–13.7, *p* = 0.026) and LDH (OR = 1.7, 95% CI: 1.1–2.7, *p* = 0.015).

**TABLE 6:** Multivariate analysis for evaluating the risk of 60-day mortality.

	OR	[CI 95%]	*p*-value
Chronic renal failure	4.02	1.18–13.72	**0.026**
LDH (Ln)	1.72	1.10–2.65	**0.015**
APACHEII > 16	2.67	1.33–5.40	**0.006**
ARDS	2.67	1.14–6.29	**0.024**

Bold values are statistically significant *p*-values.

## 4 Discussion

In this cohort of 454 consecutive post-operative patients with sepsis or septic shock, the most relevant findings were: (i) 10% developed ARDS; (ii) higher SOFA score and higher levels of lactate were independent risk factors for ARDS development; (iii) ARDS was associated with prolonged ICU stay and duration of MV; (iv) the presence of ARDS increases mortality by 2.7-fold; and (v) chronic renal failure, elevated LDH, and higher APACHE score were independently associated with 60-day mortality.

Prevalence of ARDS in septic patients ranges between 7 and 14% (21), and several reports have found a similar prevalence (16, 17, 22, 23). Although blood transfusion thresholds and MV have been modified over the last 20 years, post-operative ARDS prevalence has not declined. Lung and abdominal infections are the most frequent sources of infection in septic patients developing ARDS (12), as found in our study. A recent study (24) found that pneumonia was independently associated with ARDS development. Thus, ICU patients who develop sepsis by respiratory tract or abdominal infections, should be carefully monitored. APACHE II score, SOFA score, and elevated serum lactate have been associated with ARDS development (4, 11, 25), as in our study. Therefore, post-operative septic patients with higher severity of illness should be carefully monitored to prevent ARDS development. Of note, monitoring serial lactate in patients with sepsis or septic shock seems to be associated with a decrease in major pulmonary complications, including ARDS, although more research is needed in the ICU field (26). The presence of shock, direct lung injury (including pneumonia and aspiration), and severe sepsis are also risk factors for sepsis-induced ARDS (27).

PaO_2_/FiO_2_ at baseline is not an acceptable predictor for mortality (28). Although we found that lower PaO_2_/FiO_2_ at baseline and at 48 h were associated to ARDS development, only PaO_2_/FiO_2_ at 48 h was associated with mortality. Although no particular type of surgery was significantly associated to ARDS development, the vast majority of patients who developed ARDS had surgery as a result of acute abdominal infection (11, 24). Thus, although emergency surgery could be an ARDS developing risk factor, emergency surgery was a risk factor for post-operative sepsis (29, 30) in our study. We found that age and cancer were not risk factors for ARDS development, despite that some authors (22, 23) reported that both factors are associated with ARDS development. As opposed to our study, chronic pulmonary disease (25) and chronic kidney failure (21) have been reported to predispose to ARDS. Bellani et al. (1) found that ICU length of stay was significantly associated with ARDS, but hospital stay was not significantly increased in our study. Since ICU stay, hospital stay, and duration of MV stay were associated with morbidity, those variables could be considered modifiable factors to increase survival.

Sepsis-induced ARDS mortality ranges widely between 20 and 50% (21, 25), whereas the fatality rate is about fourfold higher when compared to those septic patients without ARDS (4, 31). Moreover, ARDS recovery is more challenging when it is caused by sepsis (22, 32). Therefore, a rapid diagnose and risk factors recognition could be of paramount importance to start ARDS protective treatment as quickly as feasible. Bellani et al. (1) reported a 40% mortality in their ARDS population during hospital admission, while we found a 51% mortality during the same period. In our septic population, those who developed ARDS doubled the mortality of those post-operative septic patients without ARDS. However, most deaths in ARDS patients were not directly related to lung damage but to extrapulmonary multisystem organ failure (23), which relates to the independent association to mortality of chronic renal failure and high general status, as supported by the APACHE II score. We believe that there are critical differences in mortality depending on the ARDS severity (1); therefore, grading patients following the Berlin criteria (14) could be helpful to predict the true risk for death. Recently published ESICM guidelines (33) have reported that mortality could change between ARDS sub-phenotypes, and that multiple system organ failure, acidosis, or hyper-inflammatory response are associated with higher mortality. Since SARS-CoV-2 pandemic, several trials have examined a variety of new treatments, but long-term effects persist unknown (34), so ARDS prevention still seems the best option. Nevertheless, conducting randomized studies to evaluate ARDS and mortality risk factors poses challenges, with establishing causal inference proving to be a crucial consideration, as causal associations can have major impact in clinical management (35).

We acknowledge that our study has potential limitations. First, due to the retrospective nature of our study design, there is a possibility that some patients were misclassified based on our available radiographs and blood gas measurements. However, we based the definition of ARDS on the Berlin criteria (14) and verified the accuracy of our assessment by a separate case review by an independent physician investigator. Second, a larger population would be needed to confirm that other variables could be associated with ARDS development. Third, further studies should be conducted to assess whether prevalence of ARDS has declined due to improvements in transfusion limits and MV strategies.

In summary, post-operative sepsis-induced ARDS was associated with higher 60-day mortality compared to non-ARDS post-operative septic patients. Post-operative septic patients with higher severity of illness have a greater risk of ARDS and worse outcomes. Chronic renal failure, APACHE II score, and LDH were also independently associated with 60-day mortality. Further investigation should be performed on post-operative sepsis-induced ARDS to prevent its occurrence.

## Data availability statement

The data analyzed in this study is subject to the following licenses/restrictions: the datasets generated and/or analyzed during the current study are available from the corresponding authors on reasonable request. Requests to access these datasets should be directed to RL-H, rocio.lopez.herrero@uva.es.

## Ethics statement

The studies involving humans were approved by the Ethics Committee for Clinical Research, Hospital Clínico Universitario de Valladolid, Valladolid, Spain. The studies were conducted in accordance with the local legislation and institutional requirements. The participants provided their written informed consent to participate in this study.

## Author contributions

MB-C: Conceptualization, Data curation, Formal Analysis, Investigation, Methodology, Writing – original draft, Writing – review and editing. MM-F: Data curation, Formal Analysis, Methodology, Writing – original draft, Writing – review and editing. RL-H: Conceptualization, Investigation, Methodology, Writing – review and editing. JP-V: Data curation, Formal Analysis, Investigation, Writing – review and editing. MH-R: Investigation, Methodology, Resources, Writing – review and editing. EG-S: Formal Analysis, Investigation, Writing – review and editing. EG-P: Investigation, Methodology, Resources, Writing – review and editing. ML-L: Data curation, Formal Analysis, Writing – review and editing. PJ-M: Investigation, Methodology, Resources, Writing – review and editing. RP-A: Data curation, Investigation, Writing – review and editing. JV: Funding acquisition, Methodology, Project administration, Supervision, Validation, Writing – review and editing. ET: Conceptualization, Funding acquisition, Methodology, Project administration, Resources, Supervision, Writing – original draft, Writing – review and editing.
